# Supplementary material for: Identification of photoperiod-induced specific miRNAs in the adrenal glands of Sunite sheep (Ovis aries)
Source: Front Vet Sci. 2022 Jul 22;9:888207. doi: 10.3389/fvets.2022.888207 (PMC9354845; doi:10.3389/fvets.2022.888207)
Supplement: Supplementary file 1 [file Data_Sheet_1.ZIP › Supplementary file/miRDeep2 file.zip!/miRDeep2 file/Know_Structure/SPLP42A2/oar-mir-154a.pdf]

miRBase precursor : oar-mir-154a
Total read count : 1461
oar-miR-154a-5p read count : 21
oar-miR-154a-3p read count : 1439
remaining reads : 1

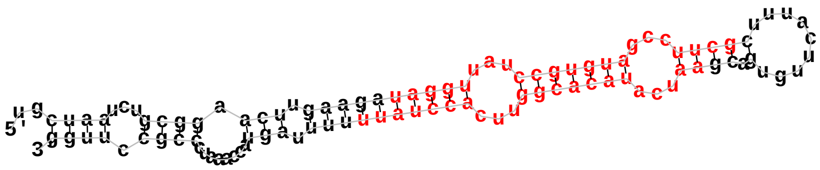

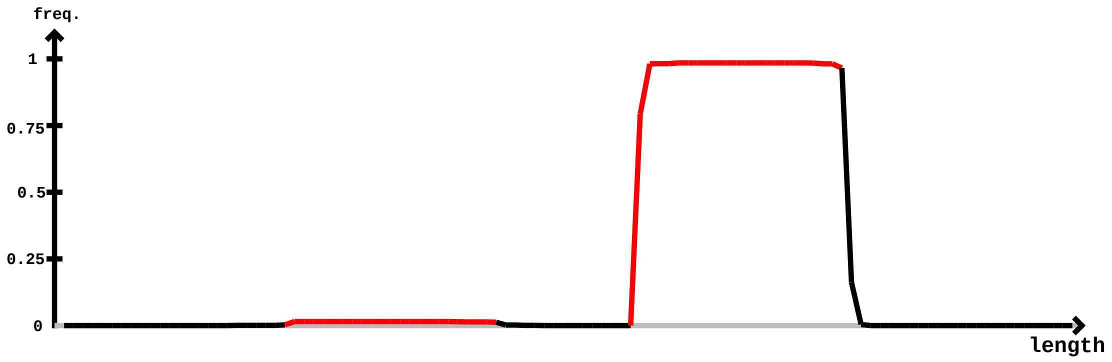

oar-miR-154a-5p

oar-miR-154a-3p

```
5'- ugcuaaucugcggaacuugaagauagguuauccguguagccuucgcuuuacuugugacgaaucauacacgguucaccuauuuuuuaguaccaaaucccgccuugg   -3'    exp
    .((......))((.(((.((((((((((...(((((((...(((((.........).))))...)))))))...)))))))))).))).)).............    reads    mm    sample
    .................ugaagauagguuauccguguagc.................................................................    1        0     seq
    ......................auagguuauccguguagcc................................................................    1        0     seq
    ......................auagguuauccguguagccuuc.............................................................    1        0     seq
    ......................auagguuauccguguagccuucg............................................................    1        0     seq
    .......................uagguuauccguguagccuuc.............................................................    1        0     seq
    .......................uagguuauccguguagccuucg............................................................    14       0     seq
    .......................uagguuauccguguagccuucgcu..........................................................    2        0     seq
    .......................uagguuauccguguagccuucgcuuu........................................................    1        0     seq
    ...........................................................aaucauacacgguucaccu...........................    3        0     seq
    ...........................................................aaucauacacgguucaccuau.........................    22       0     seq
    ...........................................................aaucauacacgguucaccuauu........................    1118     0     seq
    ...........................................................aaucauacacgguucaccuauuu.......................    16       0     seq
    ............................................................aucauacacgguucacc............................    1        0     seq
    ............................................................aucauacacgguucaccu...........................    1        0     seq
    ............................................................aucauacacgguucaccuau.........................    1        0     seq
    ............................................................aucauacacgguucaccuauu........................    52       0     seq
    ............................................................aucauacacgguucaccuauuu.......................    214      0     seq
    ............................................................aucauacacgguucaccuauuuu......................    6        0     seq
    .............................................................ucauacacgguucaccuauuu.......................    1        0     seq
    ...............................................................auacacgguucaccuauu........................    4        0     seq
```
